# Evaluation of the 7^th^ and 8^th^ editions of the AJCC/UICC TNM staging systems for lung cancer in a large North American cohort

**DOI:** 10.18632/oncotarget.18158

**Published:** 2017-05-24

**Authors:** Lin Yang, Shidan Wang, Yunyun Zhou, Sunny Lai, Guanghua Xiao, Adi Gazdar, Yang Xie

**Affiliations:** ^1^ Department of Pathology, National Cancer Center/Cancer Hospital, Chinese Academy of Medical Sciences and Peking Union Medical College, Beijing, China; ^2^ Department of Clinical Sciences, Quantitative Biomedical Research Center, University of Texas Southwestern Medical Center, Dallas, Texas, USA; ^3^ Department of Data Science, University of Mississippi Medical Center, Jackson, Mississippi, USA; ^4^ Hamon Center for Therapeutic Oncology, University of Texas Southwestern Medical Center, Dallas, Texas, USA; ^5^ Department of Pathology, University of Texas Southwestern Medical Center, Dallas, Texas, USA

**Keywords:** non-small cell lung cancer, AJCC/UICC staging system, national cancer database, survival analysis, external validation

## Abstract

**Purpose:**

The new 8^th^ American Joint Committee on Cancer (AJCC)/International Union for Cancer Control (UICC) lung cancer staging system was developed and internally validated using the International Association for the Study of Lung Cancer (IASLC) database, but external validation is needed. The goal of this study is to validate the discriminatory ability and prognostic performance of this new staging system in a larger, independent non-small cell lung cancer (NSCLC) cohort with greater emphasis on North American patients.

**Methods:**

A total of 858,909 NSCLC cases with one malignant primary tumor collected from 2004 to 2013 in the National Cancer Database (NCDB) were analyzed. The primary coding guidelines of the Collaborative Staging Manual and Coding Instructions for the new 8^th^ edition AJCC/UICC lung cancer staging system was used to define the new T, M and TNM stages for all patients in the database. Kaplan-Meier curves, Cox regression models and time-dependent receiver operating characteristics were used to compare the discriminatory ability and prognostic performance of the 7^th^ and the revised 8^th^ T, M categories and overall stages.

**Results:**

We demonstrated that the 8^th^ staging system provides better discriminatory ability than the 7^th^ staging system and predicts prognosis for NSCLC patients using the NCDB. There were significant survival differences between adjacent groups defined by both clinical staging and pathologic staging systems. These staging parameters were significantly associated with survival after adjusting for other factors.

**Conclusions:**

The updated T, M, and overall TNM stage of the 8^th^ staging system show improvement compared to the 7^th^ edition in discriminatory ability between adjacent subgroups and are independent predictors for prognosis.

## INTRODUCTION

Lung cancer is one of the most common malignant tumors in the world and the leading cause of cancer-related death worldwide [1]. Although considerable progress has been made in the screening of early lung cancer and in applying targeted therapy to treat advanced cancers, the prognosis of lung cancer remains poor [2, 3]. Accurate categorization of the tumor stage is crucial for prognostic assessment and determining the stage-specific therapeutic strategy. The American Joint Committee on Cancer (AJCC)/International Union for Cancer Control (UICC) tumor, node, and metastasis (TNM) staging system for lung cancer has been revised from the 5^th^ to the 8^th^ editions over the last two decades, and the 8^th^ edition will be instituted in January 2018. As compared to the 7^th^ edition, the 8^th^ edition staging system introduced changes to classification in both the T and M categories as well as in the overall stage grouping [4–6].

For the purposes of developing the newly revised 8^th^ edition of TNM lung cancer staging system, a new database was collected by the International Association for the Study of Lung Cancer (IASLC). The database contained 94,708 cases of patients diagnosed with lung cancer from 1999 to 2010, donated from 35 sources in 16 countries [7], and was used for both the development and the internal validation of the 8^th^ edition of the staging system. However, external validation of the 8^th^ edition of the classification is necessary. For the 7^th^ edition, external validation was performed using the North American Surveillance, Epidemiology, and End Results Registries (SEER) database [4]. External validation of the proposed changes made by the 8^th^ edition could not be performed with the SEER database due to the limited availability of certain site-specific factors. In addition, only 5% (N=4,660) of patients in the IASLC database for the 8^th^ staging system were from North America. In comparison, 21% (N=21,130) of patients in the IASLC database developed for the 7^th^ edition were from North America. Factors beyond anatomic characteristics of the disease have important implications on prognosis, including geographic region, time period, and type of database. Since North American patients are relatively underrepresented in the 8^th^ edition, external validation in a large North American cohort is especially important. The National Cancer Database (NCDB) includes over 1.1 million patients diagnosed with non-small cell lung cancer (NSCLC) from 2004 to 2013 in the United States, providing an ideal external dataset to validate the discriminatory and prognostic performance of the 8^th^ edition lung cancer staging system. The goal of this study is to evaluate the discriminatory ability of the revised 8^th^ edition T category, M category and overall stages as well as the prognostic accuracy of the staging system and compare it with the 7^th^ edition staging system in the NCDB cohort.

## RESULTS

### Evaluation of the T component

**Figure 1:**
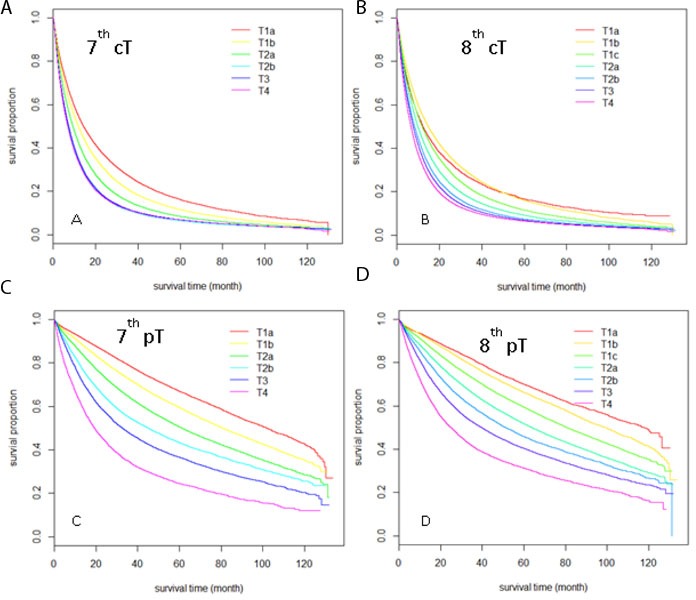
Kaplan-Meier Survival curves comparison among different T stages sub-groups Sub-groups were defined by clinical T stage according to the 7^th^ edition **A.**, and the 8^th^ edition **B.**; sub-groups were defined by pathological T stage according to the 7^th^ edition **C.** and 8^th^ edition **D.**

A total of 368,367 cases with cT and 177,409 cases with pT parameters were included in the comparison of the 7^th^ and 8^th^ edition T categories. Patient numbers in different subgroups according to the 7^th^ and 8^th^ edition subdivision guidelines were cross-tabulated in Table 1. As the table shows, changes occurred mainly in the following categories: the 7^th^ edition T1a was divided into the 8^th^ edition T1a & T1b categories; T2a was divided into T2a and T2b categories; and T3 category was re-categorized into T2, T3 and T4 groups. To see how these changes affected the overall survival (OS) outcomes, we drew Kaplan-Meier curves for OS for the subgroups of the T category of the 7^th^ and 8^th^ editions based on cT staging (Figure 1A, 1B) and pT staging (Figure 1C, 1D) separately, and also performed univariate Cox regression analysis between all pairs of adjacent subgroups in the 7^th^ and 8^th^ edition cT and pT subgrouping (Table 2). For pT staging parameters, the 8^th^edition staging system has better discriminatory ability compared to the 7^th^edition staging system, and the survival time decreases as the pathological stage progresses to the next sub-groups for all sub-groups. For example, the median survival time (MST) for the newly defined pT1b vs. pT1a group was 99.1 months vs. 117.8 months (HR=1.16, p<0.001). For cT staging parameters, the improvement in discriminatory ability appears mainly for the advanced stages, but the survival curves overlap for early stages (for example, cT1b vs. cT1a in the 8^th^ edition in Figure 1B), and the median survival time is longer for T1b (14.7 vs. 12.3 months). For the overall performance between the 8^th^ and 7^th^ editions of T staging, the C-index for the 8^th^ pT subgrouping (0.610±0.001) was higher than that of the 7^th^ edition (0.608±0.001), and a similar result was found in the 8^th^ edition cT subgrouping (0.556±0.001) compared to that of the 7^th^ edition cT (0.551±0.001).

**Table 1:** Cross tables for patients included for analysis of T stages (the 7^th^ edition *vs*. the 8^th^ edition)

Clinical T stage		7^th^ edition					
		T1a	T1b	T2a	T2b	T3	T4
**8**^th^ **edition**	T1a	6,261	-	-	-	-	-
	T1b	36,380	-	-	-	-	-
	T1c	-	47,705	-	-	-	-
	T2a	-	-	63,136	-	-	-
	T2b	-	-	38,735	-	2,013	-
	T3	-	-	-	44,645	36,843	-
	T4	-	-	-	-	33,527	55,478
**Pathological T stage**		**7**^th^ **edition**					
T1a	T1b	T2a	T2b	T3	T4
**8**^th^ **edition**	T1a	9,599	-	-	-	-	-
	T1b	41,999	-	-	-	-	-
	T1c	-	32,123	-	-	-	-
	T2a	-	-	44,387	-	758	-
	T2b	-	-	13,654	-	289	-
	T3	-	-	-	12,650	11,096	-
	T4	-	-	-	-	7,088	3,856

**Table 2:** Univariate Cox regression analysis result for T stage in the 7^th^& 8^th^ editions, both for clincial T(cT)and pathological T(pT)(MST, median survival time)

7^th^cT	*N*	Events	MST(m)	HR	*P*-value
T1a	42,641	32,358	14.4	-	
T1b	47,705	38,626	11.9	T1b vs T1a: 1.18	< 0.001
T2a	10,187	86,875	9.3	T2a vs T1b: 1.20	< 0.001
T2b	44,645	39,186	7.2	T2b vs T2a: 1.20	< 0.001
T3	76,027	67,485	7.3	T3 vs T2b: 0.98	<0.001
T4	55,478	48,908	6.9	T4 vs T3: 1.03	< 0.001
**8**^th^ **cT**	***N***	**Events**	**MST(m)**	**HR**	***P*-value**
T1a	6,261	4,766	12.3	-	-
T1b	36,380	27,592	14.7	T1b vs T1a: 0.94	< 0.001
T1c	47,705	38,626	11.9	T1c vs T1b: 1.19	< 0.001
T2a	66,780	56,561	9.9	T2a vs T1c: 1.14	< 0.001
T2b	40,748	35,267	8.3	T2b vs T2a: 1.13	< 0.001
T3	81,488	71,858	7.7	T3 vs T2b: 1.06	< 0.001
T4	89,005	78,768	6.6	T4 vs T3: 1.09	< 0.001
**7**^th^**pT**	***N***	**Events**	**MST(m)**	**HR**	***P*-value**
T1a	51,598	16,239	101.8	-	-
T1b	32,123	12,566	80.6	T1b vs. T1a:1.30	< 0.001
T2a	57,951	27,319	61.2	T2a vs T1b: 1.30	< 0.001
T2b	12,650	6,827	44.9	T2b vs T2a: 1.26	< 0.001
T3	19,231	11,814	32.6	T3 vs T2b: 1.22	< 0.001
T4	3,856	2,776	19.2	T4 vs T3: 1.45	< 0.001
**8**^th^ **pT**	***N***	**Events**	**MST(m)**	**HR**	***P*-value**
T1a	9,599	2,703	117.8	-	-
T1b	41,999	13,536	99.1	T1b vs. T1c: 1.16	< 0.001
T1c	32,123	12,566	80.6	T1c vs T1b: 1.27	< 0.001
T2a	45,145	20,818	63.9	T2a vs T1c: 1.26	< 0.001
T2b	13,853	7,106	51.2	T2b vs T2a: 1.18	< 0.001
T3	23,746	13,633	39.7	T3 vs T2b: 1.20	< 0.001
T4	10,941	7,179	24.9	T4 vs T3: 1.35	< 0.001

### Evaluation of the N component

A total of 567,844 patients with pN parameters and 208,752 patients with cN parameters were included in the OS for the 7^th^ and 8^th^ editions as well. No changes have been made for N component in the 8^th^ edition compared to the 7^th^ edition. Significant difference between N0, N1, N2, N3 were found both in cN and pN subgroups (Supplementary Table 1). The C-index was 0.634±0.001 for the 7^th^/8^th^ edition pN, and 0.607±0.001 for the 7^th^/8^th^ edition cN.

### Evaluation of the M component

**Figure 2:**
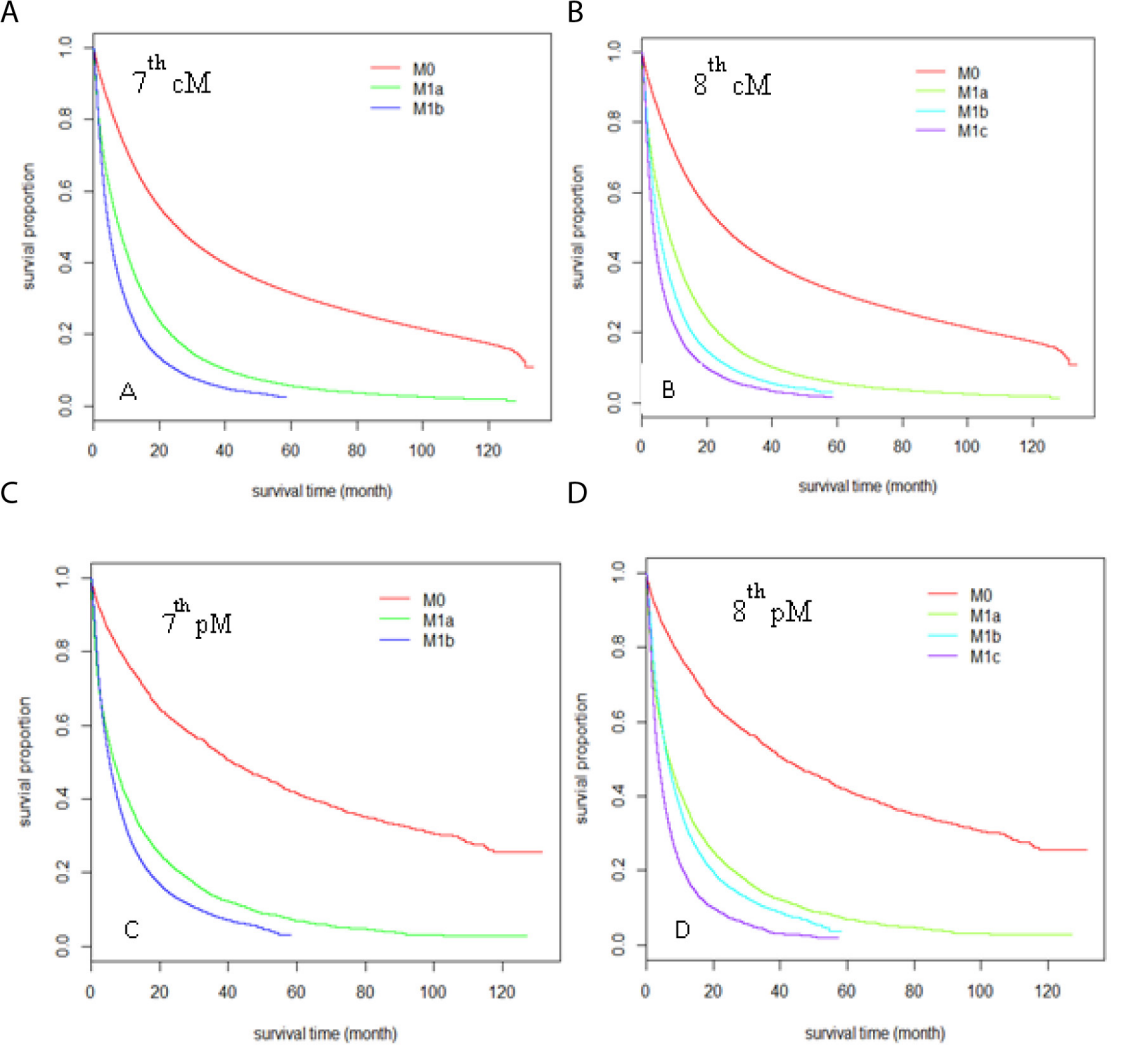
Kaplan-Meier survival curves comparison among different M stages sub-groups Sub-groups were defined by clinical M stage according to the 7^th^ edition **A.**, and the 8^th^ edition **B.** sub-groups were defined by pathological M stage according to the 7^th^ edition **C.** and 8^th^ edition **D.**

A total of 493,829 cases with cM and 24,721 cases with pM parameters were included in the comparison of the 7^th^ and 8^th^ edition M categories. Patient numbers in different subgroups according to the 7^th^ and 8^th^ subdivision guidelines were cross-tabulated in Table 3. The difference between the two editions is that the M1b stage was further refined into the M1b and M1c subgroups. Results showed the Kaplan-Meier curves for overall survival (OS) for subgroups of the M category of the 7^th^ and 8^th^ editions (Figure 2), and significant difference were found by a univariate Cox regression analysis between all pairs of adjacent subgroups in the 7^th^ and 8^th^ edition cM and pM subgrouping (Table 4). The MST for pM1c vs. pM1b is 3.5 vs. 6.3 months (HR=1.49), and the MST for cM1c vs. cM1b is 3.5 vs. 5.2 months. These show that the newly defined M subgroups in the 8^th^ edition have good discriminative ability for survival outcome. Furthermore, the C-index for the 8^th^ edition pM subgrouping (0.564±0.002) was higher than that of the 7^th^ edition (0.547±0.002), and a similar result was found in the 8^th^ edition cM subgrouping (0.581±0.001) compared to that of the 7^th^ edition cM (0.580±0.000).

**Table 3:** Cross tables for patients included for analysis of M stages(the 7^th^ edition *vs*. the 8^th^ edition), both for clinical and pathological M stages

Clinical M stage		7^th^ edition		
		M0	M1a	M1b
**8**^th^ **edition**	M0	1,409,366	-	-
	M1a	-	41,577	-
	M1b	-	-	31,283
	M1c	-	-	11,603
**Pathological M stage**		**7**^th^ **edition**		
		**M0**	**M1a**	**M1b**
**8**^th^ **edition**	M0	1,682	-	-
	M1a	-	7,775	-
	M1b	-	-	10,941
	M1c	-	-	4,323

**Table 4:** Univariate Cox regression analysis result for M stage in the 7^th^& 8^th^ editions, both for clincial M(cM)and pathological M(pM)(MST, median survival time)

7^th^cM	*N*	Events	MST(m)	HR	*P*-value
**M0**	409,366	261,531	25.3	-	-
**M1a**	41,577	36,040	7.6	M1a vs M0: 2.53	< 0.001
**M1b**	42,886	37,649	4.6	M1b vs M1a: 1.38	< 0.001
**8**^th^ **cM**	***N***	**Events**	**MST(m)**	**HR**	***P*-value**
**M0**	409,366	261,531	25.3	-	-
**M1a**	41,577	36,040	7.7	M1a vs. M0: 2.53	< 0.001
**M1b**	31,283	27,123	5.2	M1b vs M1a: 1.30	< 0.001
**M1c**	11,603	10,526	3.5	M1c vs M1b: 1.28	< 0.001
**7**^th^**pM**	***N***	**Events**	**MST(m)**	**HR**	***P*-value**
**M0**	1,682	1,106	41.2	-	-
**M1a**	7,775	6,404	6.7	M1a vs M0: 3.13	< 0.001
**M1b**	15,264	13,107	5.3	M1b vs M1a: 1.21	< 0.001
**8**^th^ **pM**	***N***	**Events**	**MST(m)**	**HR**	***P*-value**
**M0**	1,682	1,106	41.2	-	-
**M1a**	7,775	6,404	6.7	M1a vs M0:3.13	< 0.001
**M1b**	10,941	9,175	6.3	M1b vs M1a: 1.10	< 0.001
**M1c**	4,323	3,932	3.5	M1c vs M1b: 1.49	< 0.001

### Evaluation of the TNM stage grouping and prognostic accuracy

**Figure 3:**
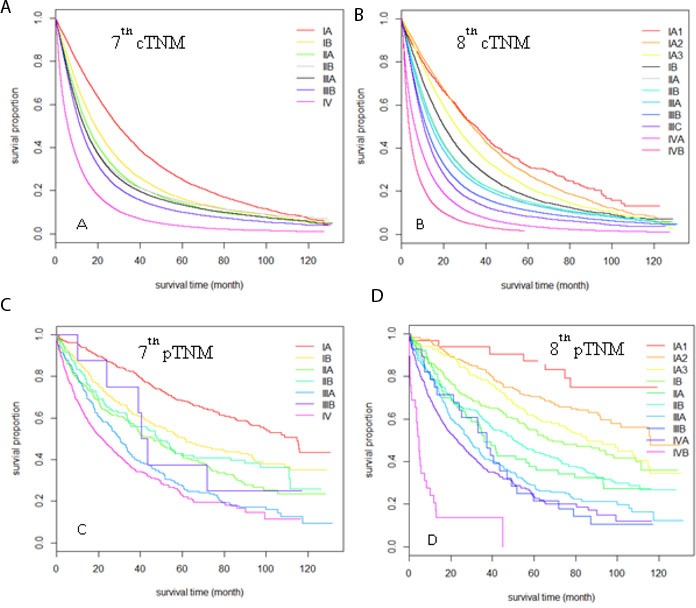
Kaplan-Meier survival curves comparison among different TNM stages sub-groups Sub-groups were defined by clinical TNM stage according to the 7^th^ edition **A.**, and the 8^th^ edition **B.** sub-groups were defined by pathological TNM stage according to the 7^th^ edition **C.** and 8^th^ edition **D.**

A total of 84,076 cases with cTNM and 1,812 cases with pTNM parameters were included in the comparison of the 7^th^ and 8^th^ edition TNM categories. Patient numbers in different subgroups according to the 7^th^ and 8^th^ edition subdivision guidelines were cross-tabulated in Table 5. Of note, the NCDB requires that reporting of any cell size<11 be suppressed, and the associated total number also be suppressed, thus we used “*” or “#” to indicate those cells in both Tables 5 and 6. The main differences between the two editions were that the 7^th^ edition stage IA was refined to IA1, IA2 and IA3, and stage IV was refined to IVA and IVB. For both pTNM and cTNM, the new stage IVB subgroup had significantly worse survival compared to stage IVA (MST: 3.6 vs. 6.3m for cIVB vs. cIVA, HR=1.5; MST: 4.7 vs. 23.0m for pIVB vs. pIVA, HR=3.18). For pathological stage IA, there was clear separation among stages pIA1, pIA2 and pIA3 (Figure 3). For clinical stage IA, the survival outcomes between cIA1 and cIA2 were similar but cIA3 had significantly worse survival outcome. This was consistent with clinical T staging results. For both clinical and pathological stages, the refined stage II and III subgroups in the 8^th^ edition had better survival discrimination than the 7^th^ edition. Due to the relatively small sample size, the variation for pTNM stage was large and the discrimination within stage II and III subgroups was still not very good.

**Table 5:** Cross tables for patients included for analysis of TNM stages (the 7^th^ edition *vs*. the 8^th^ edition) in clinical TNM(cTNM) and pathological TNM(pTNM) (* indicating numbers<11 are suppressed according to the NCDB requriement)

cTNM stage		7^th^ edition						
		IA	IB	IIA	IIB	IIIA	IIIB	IV
**8**^th^ **edition**	IA1	161	-	-	-	-	-	-
	IA2	819	-	-	-	-	-	-
	IA3	919	-	-	-	-	-	-
	IB	-	1,234	-	76	-	-	-
	IIA	-	608	-	42	-	-	-
	IIB	-	-	1,625	968	50	-	-
	IIIA	-	-	-	887	4,955	-	-
	IIIB	-	-	-	-	1,655	1,820	-
	IIIC	-	-	-	-	-	661	-
	IVA	-	-	-	-	-	-	57,776
	IVB	-	-	-	-	-	-	9,820
**pTNM stage**		**7**^th^ **edition**						
	**IA**	**IB**	**IIA**	**IIB**	**IIIA**	**IIIB**	**IV**
**8**^th^**edition**	IA1	34	-	-	-	-	-	-
	IA2	165	-	-	-	-	-	-
	IA3	140	-	-	-	-	-	-
	IB	-	161	-	*	-	-	-
	IIA	-	63	-	*	-	-	-
	IIB	-	-	171	57	*	-	-
	IIIA	-	-	-	47	135	-	-
	IIIB	-	-	-	-	20	*	-
	IIIC	-	-	-	-	-	-	-
	IVA	-	-	-	-	-	-	768
	IVB	-	-	-	-	-	-	29

**Table 6:** Univariate Cox regression analysis result for TNM stage in the 7^th^& 8^th^ editions, both for clinical & pathological TNM stages separately

7^th^cTNM	*N*	events	MST(m)	HR	*P*-value
**IA**	23,531	14,626	29.6	-	-
**IB**	15,712	11,625	18.9	IB vs IA: 1.44	<0.001
**IIA**	12,239	9,527	15.2	IIA vs IB: 1.16	<0.001
**IIB**	11,225	8,865	13.4	IIB vs IIA: 1.05	<0.001
**IIIA**	62,045	49,851	13.5	IIIA vs IIB: 1.02	<0.04
**IIIB**	29,763	24,911	11.6	IIIB vs IIIA: 1.14	<0.001
**IV**	67,596	59,133	5.8	IV vs IIIB: 1.62	<0.001
**8**^th^**cTNM**	***N***	**events**	**MST(m)**	**HR**	***P*-value**
**IA1**	1443	799	35.4	-	-
**IA2**	10,616	6,279	33.7	IA2 vs IA1:1.1	0.11
**IA3**	11,472	7,548	26.1	IA3 vs IA2: 1.2	< 0.001
**IB**	10,952	8,003	20.2	IB vs IA3: 1.2	< 0.001
**IIA**	5,503	4,200	16.0	IIA vs IB: 1.2	< 0.001
**IIB**	17,644	13,713	15.5	IIB vs IIA: 1.0	< 0.001
**IIIA**	46,822	37,083	14.3	IIIA vs IIB: 1.1	< 0.001
**IIIB**	41,140	34,121	11.7	IIIB vs IIIA: 1.2	< 0.001
**IIIC**	8,923	7,659	10.6	IIIC vs IIIB: 1.1	< 0.001
**IVA**	57,776	50,236	6.3	IVA vs IIIC: 1.4	< 0.001
**IVB**	9,820	8,897	3.6	IVB vs IVA: 1.5	< 0.001
**7**^th^ **pTNM**	***N***	**events**	**MST(m)**	**HR**	***P*-value**
**IA**	339	141	114.8	-	-
**IB**	224	130	61.1	IB vs IA: 1.66	< 0.001
**IIA**	171	118	46.3	IIA vs IB: 1.37	0.01
**IIB**	#	67	48.0	IIB vs IIA: 0.84	0.25
**IIIA**	#	137	30.1	IIIA vs IIB: 1.67	< 0.001
**IIIB**	#	*	42.0	IIIB vs IIIA: 0.66	0.32
**IV**	797	506	21.8	IV vs IIIB: 1.71	0.19
**8**^th^ **pTNM**	***N***	**events**	**MST(m)**	**HR**	***P*-value**
**IA1**	34	*	-	-	-
**IA2**	165	64	116.0	IA2 vs IA1:1.87	0.12
**IA3**	140	70	85.3	IA3 vs IA2: 1.42	0.04
**IB**	#	91	69.0	IB vs IA3: 1.18	0.29
**IIA**	#	44	36.2	IIA vs IB: 1.47	0.04
**IIB**	#	155	50.3	IIB vs IIA: 0.92	0.62
**IIIA**	182	143	29.3	IIIA vs IIB: 1.45	<0.001
**IIIB**	#	25	37.3	IIIB vs IIIA: 1.08	0.71
**IIIC**	-	-	-	-	-
**IVA**	768	480	23.0	IVA vs IIIB: 1.12	0.58
**IVB**	29	26	4.7	IVB vs IVA: 3.18	<0.001

(* indicating suppressed numbers for cell size<11 or # indicating the total number from suppressed cell size in Table 5, according to the requirment of the NCDB)(MST,median survival time).

**Figure 4:**
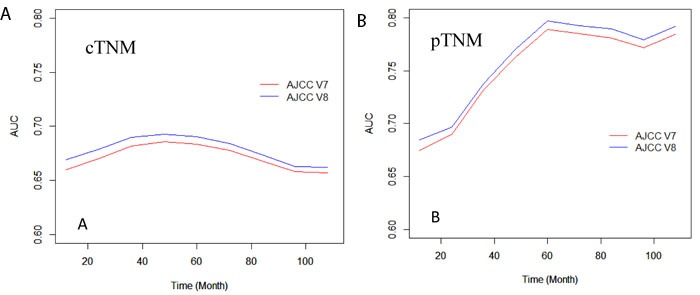
Time-dependent area-under-curves (AUC) from the 12^th^ month to 108^th^ month, calculated by the 7^th^ and 8^th^ clinical **(A)** or pathological **(B)** TNM stage, separately.

Multivariate Cox regression analysis was performed for validating the prognostic significance of the 7^th^ and 8^th^ edition TNM staging system, adjusted by age, gender, race, comorbidity, and histology. Our results were similar to the univariate analysis. Furthermore, the C-index for the 8^th^ edition pTNM subgrouping (0.644±0.009) was higher than that of the 7^th^ edition (0.636±0.009), and a similar result was found in the 8^th^ edition cTNM subgrouping (0.624±0.001) compared to that of the 7^th^ edition cTNM (0.617±0.001). Also, time-dependent ROC was calculated at 12-month intervals from the 12^th^ to the 108^th^ month (Figure 4). The average AUC of the 8^th^ edition pTNM (0.760) was higher than that of the 7^th^ edition pTNM (0.752), and the average AUC of the 8^th^ edition cTNM (0.678) was also higher than that of the 7^th^ edition cTNM (0.671), which indicates a better prognostic performance of the 8^th^ edition AJCC TNM staging system compared to that of the 7^th^ edition.

## DISCUSSION

We used the NCDB data as an external validation source for the 8^th^ edition TNM lung cancer staging system and did a comparative analysis with the pre-existing 7^th^ edition. We chose the NCDB because it is a nationally recognized clinical oncology database sourced from hospital registry data that are collected in more than 1,500 Commission on Cancer (CoC)-accredited facilities all over the United States, which represent more than 70 percent of newly diagnosed cancer cases nationwide and more than 34 million historical records [8]. It is powerfully representative of real clinical practice settings. The results of the current study showed good external validation of the 8^th^ edition staging system through comparison with the 7^th^ edition, focusing on the discriminative ability of each adjacent subgroup of the newly revised T and M components and overall stage grouping.

The newly revised 8^th^edition TNM staging system introduced changes in the classification of the T descriptor and M descriptor, but introduced no changes in N classification similar to that of the 7^th^ edition. The revision for the 8^th^ edition compared to the 7^th^ edition of AJCC TNM staging system consisted of changes in the T descriptors that reclassifies tumor size into more redefined T subgroups, reclassifies tumor involvement in the main bronchus regardless of distance from carina, reclassifies atelectasis/pneumonitis, reclassifies diaphragm invasion and deletes mediastinal pleural effusion as a T descriptor [9]. Benefitting from the detailed coding system of NCDB, all of the above T descriptor related factors were included in definitions and analysis for this comparison and analysis study. It was found that the 8^th^ edition T categorization schema performs better than that of the 7^th^ edition in discriminating different T subgroups, especially in the pT1 stage. The subdivision of T1 in the 8^th^ edition will offer greater prognostic precision in critical points of making surgical decisions. For example, lobectomy, as compared to sublobar resection, was traditionally considered the procedure of choice for early stage lung cancer due to lower rates of local recurrence [10, 11]. More recent studies indicate that segmentectomy may be sufficient in certain populations [5]. As Phase III trials are initiated to better define the role of sublobar resection for high-risk operable patients with NSCLC ≤ 3cm, the greater degree of subgrouping in the 8^th^ edition may prove to be valuable in categorizing small, early-stage tumors in a way that mirrors the decision between sublobar resection and lobectomy [11]. The survival curves overlap for clinical T1b vs. T1a using the 8^th^ edition clinical TNM IA1 and IA2. The reason clinical staging may not be as discriminative as pathological staging is the lower precision of tumor size measurement for very small tumors. In particular, lung adenocarcinomas (≤3 cm) with lepidic histology typically have the appearance of ground glass opacity (GGO), which is generally not recorded within the tumor size, according to the 8^th^ edition T descriptor classification of small lung adenocarcinomas with a GGO and lepidic component by CT and pathologic assessment [12]. This needs to be further studied for the refinement of T1a and T1b stage in the 8^th^ edition.

The present study has several limitations. First, because this is a retrospective analysis we have no access to information for subdividing Tis and T1a (mi) according to the 8^th^ edition proposal, which needs to be confirmed by pathological observation. Prospective collection of appropriate cases is desirable for addressing the discrepancy between stage IA and IB. Second, the sample size was relatively small for evaluating the pathological TNM staging system, which leads to large variation in the survival estimations. Third, the comparison of “M1b” to “M1c” in our analysis was between single-organ and multi-organ metastatic involvement, rather than between oligometastatic and multi-lesion metastases as intended by the IASLC, because there was no record of metastatic sites in one or more organs. Still, a significant difference was found between “M1b” and “M1c”, despite the confounding presence of multi-lesion single organ disease in “M1b” narrowing the differences between the categories. For the same reasons as stated above for evaluation of the M descriptor, analysis was limited for the 8^th^ edition stage IV disease, as the M1b descriptor is categorized under stage IVA while the M1c descriptor is categorized under stage IVB.

In summary, this is the first external validation of the newly revised 8^th^ edition TNM staging system, based on a large North American cohort from the NCDB. Since North American patients are relatively underrepresented in the 7^th^ and 8^th^ editions, external validation in a large North American cohort is especially important. Our study validated both the 7^th^ and 8^th^ editions of staging for NSCLC using both clinical and pathological measures and concluded that the 8^th^ edition has overall better discriminative ability for overall survival outcomes.

## CONCLUSIONS

Both the 7^th^ and 8^th^ editions of staging for NSCLC, using both clinical and pathological measures, were validated in a large North American focused cohort. The updated T and M categories and overall stages of the 8^th^ staging system were better than in the 7^th^ edition in discriminatory ability for the adjacent subgroups, and were also independent predictive factors for prognosis.

## MATERIALS AND METHODS

### Patient population and inclusion criteria

The University of Texas Southwestern Medical Center Institutional Review Board approved this retrospective analysis of the National Cancer Database (NCDB) dataset. The NCDB is a jointly administered effort by the American Cancer Society and the American College of Surgeons Commission on Cancer (CoC), collecting data from more than 1,500 cancer facilities around the United States. The database currently contains more than 30 million patient records and is estimated to capture approximately 70% of all new cancer diagnoses in the United States. 1,163,465 de-identified NSCLC cases from the NCDB were collected, all of which were diagnosed from 2004 to 2013. Patients with more than one malignant primary tumor and with a tumor size larger than 10cm were excluded from the analysis, leaving 858,909 cases. Based on the primary coding dictionary of NCDB, we collected variables associated with the definition of T categories from Collaborative Stage (CS) Data Collection System, including tumor size, pleural invasion, and invasion of adjacent tissue or organs. We also collected CS Mets at Dx for bone, brain, and liver to classify M1b and M1c subgroups. For M1a and M0, there were no changes between the 7^th^ and 8^th^ editions, so we kept the primary data for analysis. Of note, these variables are only available for cases after 2010, thus M1a, M1b and M1c subgroups analysis were applied to patients diagnosed since 2010 instead of 2004.

### Staging

Using the CS tumor size, pleural invasion, and invasion of adjacent tissue or organs, and Mets at Dx variables in the NCDB database, we defined 7^th^ and 8^th^ edition clinical and pathological T/M categories according to the guidelines [5, 9, 13, 14]. Specifically, the new 8^th^ T categorization schema was grouped by 1-cm increments in tumor size up to more than 7-cm. The T1 category was subdivided into T1a (≤1cm), T1b (>1 to ≤ 2cm), and T1c (>2 to ≤ 3cm); T2 was subdivided into T2a (>3 to ≤4cm) and T2b (>4 to ≤5cm); tumors within the sizes of 5-7cm were reclassified as T3; and tumors greater than 7cm were redefined as T4[9]. The NCDB dataset contained information on sites of organ metastases, but not on the number of metastatic lesions at a particular organ. Thus in the current retrospective dataset of NCDB, patients with metastases to a single organ were assigned as the M1b descriptor. Patients who had metastases to multiple different organs were assigned the M1c descriptor. For the N descriptor, there are existing N0, N1, N2, and N3 subgroups in NCDB; we kept the primary data as a subgrouping of N category. Stage grouping was then assigned according to the 7^th^ and 8^th^ edition TNM classification proposed by the IASLC.

For the comparison of T staging criteria in the 7^th^ and 8^th^ editions, only cases with information on both the 7^th^ and 8^th^ edition T stage and survival outcome were included, and 368,267 cases with pathological T (pT) stage and 177,409 cases with clinical T (cT) stage were analyzed separately. For comparison of the M staging criteria, similarly, only cases with information on both the 7^th^ and 8^th^ edition M stage and survival outcome were included, and 24,721 cases with pathological M (pM) stage and 493,829 cases with clinical M (cM) stage were analyzed separately. The N staging criteria remained unchanged between the 7^th^ and 8^th^ edition, and 567,844 cases were included in the pathological N (pN) group and 208,752 cases in clinical N (cN) group for the analysis. For overall TNM staging criteria for both the 7^th^ and 8^th^ editions, only those cases with all information on separate T, N, and M parameters were included in the analysis. 1,812 cases with pathological TNM (pTNM) parameters and 84,076 cases with clinical TNM (cTNM) parameters were collected.

### Statistical analysis

Overall survival data was measured from the date of diagnosis to the date of death or last contact. Kaplan-Meier survival curves, univariate and multivariate Cox regression models, and Wald tests were used to compare the discriminatory ability of the 7^th^ and the revised 8^th^ edition T category, M category and overall stages. All statistical results were considered significant if p-value ≤ 0.05. The concordance index (C-index) and time-dependent receiver operating characteristics (ROC) were used to compare the prognostic accuracy of the 7^th^ and 8^th^ edition staging schemas. The area under the curve (AUC) of ROC was calculated at 12-month intervals from the 12^th^ to the 108^th^ month for both the 7^th^ and 8^th^ edition staging systems. The time-dependent AUC curve was calculated by Inverse Probability of Censoring Weighting (IPCW) estimation. All analyses were performed in R software, V3.3.2. The R packages “survival” and “timeROC” were used.

## SUPPLEMENTARY MATERIALS FIGURE AND TABLE


