# Supplementary material for: Evaluation of the 7th and 8th editions of the AJCC/UICC TNM staging systems for lung cancer in a large North American cohort
Source: Oncotarget. 2017 May 24;8(40):66784–95. doi: 10.18632/oncotarget.18158 (PMC5620136; doi:10.18632/oncotarget.18158)
Supplement: Supplementary file 1 [file oncotarget-08-66784-s001.pdf]

Evaluation of the 7th and 8th editions of the AJCC/UICC TNM staging systems for lung cancer in a large North American cohort

Supplementary Material

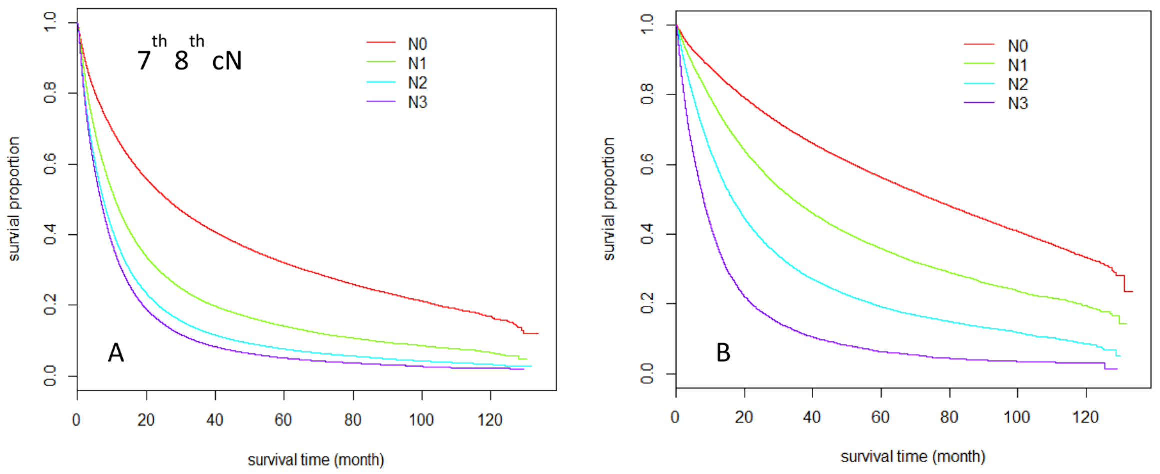

Supplementary Figure 1: Kaplan-Meier Survival curves comparison among different clinical N (cN) and pathological N (pN) stages both in the 7th edition and the 8th edition.

Supplementary Table 1: Univariate Cox regression analysis result for clinical & pathological N (cN /pN) stages(MST,median survival time).

7th/8th cN	N	Events	MST(m)	HR	P-value
N0	225,892	137,423	26	-	-
N1	56,532	45,387	11	N1 vs. N0:1.8	< 0.001
N2	205,215	178,778	8	N2 vs. N1:1.3	< 0.001
N3	80,205	72,098	7	N3 vs. N2: 1.1	< 0.001
**7th/8th pN**	**N**	**Events**	**MST(m)**	**HR**	**P-value**
N0	134,941	56,338	75	-	-
N1	30,025	18,303	34	N1 vs. N0: 1.8	< 0.001
N2	34,990	26,815	17	N2 vs. N1:1.7	< 0.001
N3	8,796	7,848	8	N3 vs. N2:1.8	< 0.001
